# Toward an Intelligent Campus: IoT Platform for Remote Monitoring and Control of Smart Buildings

**DOI:** 10.3390/s22239045

**Published:** 2022-11-22

**Authors:** Mohamed A. Ahmed, Sebastian A. Chavez, Ali M. Eltamaly, Hugo O. Garces, Alejandro J. Rojas, Young-Chon Kim

**Affiliations:** 1 Department of Electronic Engineering, Universidad Técnica Federico Santa María, Valparaíso 2390123, Chile; 2 Electrical Engineering Department, Faculty of Engineering, Mansoura University, Mansoura 35516, Egypt; 3 Sustainable Energy Technologies Center, King Saud University, Riyadh 11421, Saudi Arabia; 4 Departamento Ingeniería Informática, Universidad Católica de la Santísima Concepción, Concepción 4090541, Chile; 5 Departamento Ingeniería Eléctrica, Universidad de Concepción, Victor Lamas 1290, Concepción 4070386, Chile; 6 Department of Computer Engineering, Jeonbuk National University, Jeonju 561-756, Republic of Korea

**Keywords:** intelligent campus, smart building, internet of things platform, remote monitoring and control

## Abstract

With the growing need to obtain information about power consumption in buildings, it is necessary to investigate how to collect, store, and visualize such information using low-cost solutions. Currently, the available building management solutions are expensive and challenging to support small and medium-sized buildings. Unfortunately, not all buildings are intelligent, making it difficult to obtain such data from energy measurement devices and appliances or access such information. The internet of things (IoT) opens new opportunities to support real-time monitoring and control to achieve future smart buildings. This work proposes an IoT platform for remote monitoring and control of smart buildings, which consists of four-layer architecture: power layer, data acquisition layer, communication network layer, and application layer. The proposed platform allows data collection for energy consumption, data storage, and visualization. Various sensor nodes and measurement devices are considered to collect information on energy use from different building spaces. The proposed solution has been designed, implemented, and tested on a university campus considering three scenarios: an office, a classroom, and a laboratory. This work provides a guideline for future implementation of intelligent buildings using low-cost open-source solutions to enable building automation, minimize power consumption costs, and guarantee end-user comfort.

## 1. Introduction

The Chilean new energy efficiency law No. 21,305 was published on 13 February 2021, establishing Chile’s national energy efficiency plan. Among the main aspects of energy efficiency that the new law targets are the national energy efficiency plan, energy management of large consumers, energy labeling/rating for buildings, and efficiency standards for vehicles [1]. Considering that buildings are responsible for about 60% of the total global electricity consumption [2], information on energy usage is fundamental for the development of different energy management system (EMS) solutions [3,4,5,6,7,8,9,10,11,12,13,14,15,16,17,18,19,20,21,22,23]. In particular, such solutions are critical for those users in charge of buildings administration. Knowing the different equipment power consumptions is vital for controlling the expenses associated with building operations.

The applications of IoT in smart homes [9,10,11,12,14] and smart buildings [2,3,4,5,6,7,8,13,15,16,17,18,19,20,21,22,23] have been discussed in many publications covering different domains, including surveys [6,7,10,11,12,15,18], architectures [5], frameworks [8,14], platforms [9], and algorithms [16]. However, most of the research is based on assumptions or simulations which ignore the practical issues from the real implementation. Examples of real implementation can be found in [3,4,9,12,19]. Furthermore, the condition of the electricity system differs between countries and regions, which then requires identifying the specific requirements and needs for each design. This work aims to fill the gap related to technical implementation in a realistic environment. The main objective of this work is to design and implement an IoT platform that integrates information from various smart sensor nodes and measuring devices connected to buildings for obtaining information related to power consumption in order to support energy management solutions. All collected data will be processed to deliver the relevant information to the end user, who can then perform remote actions accordingly. The main activities required to achieve the proposed objective are:

- Define the system requirements for intelligent buildings and prioritize these requirements for developing the solution, including data collection from different sensor nodes and measurement devices.
- Build the IoT platform for smart buildings, which includes four main layers: power layer, data acquisition layer, communication network layer, and application layer.
- Implement the back-end and front-end systems. The proposed solution involves the development of a network of sensors and measurement devices and the integration of the processing unit and the databases. The front-end implementation phase consists of developing a user interface for interaction, visualization, and data analysis.
- Implementation of the testbed and validation, where the designed prototype will be installed and tested in the context of a real application (Department of Electronic Engineering, Universidad Técnica Federico Santa María (UTFSM), Valparaíso, Chile), including an office, a laboratory, and a classroom to assess the functionality and usability of the proposed solution.

This paper is structured as follows: Section 2 presents a review of related work for smart buildings. Section 3 introduces the hierarchical energy management architecture for the intelligent campus. Section 4 presents the proposed IoT architecture for smart buildings. Section 5 discusses and analyses the proposed solution and the results. Section 6 presents the conclusion and future work.

## 2. Related Work

Smart buildings and building energy management systems (BEMS) are active research areas with different application domains such as demand response programs, optimizing building power consumption, integrating renewable energy systems, etc. In [2], the authors highlighted that IoT provides a new opportunity to integrate intelligence into building management systems. Such IoT solutions are cost-effective, enabling monitoring and managing the energy consumption of the buildings. The work summarized the application of IoT in buildings, including lighting, heating, ventilation, air conditioning (HVAC), flexible loads, human detection and diagnostics, and prognostics. A case study was discussed on how to use low-cost IoT devices to provide building management with key insights into human activity and occupancy detection.

In [3], the authors presented the design and implementation of an IoT gateway for a cloud-based building energy management system. The work focused on the software architecture and the software design of the gateway device, which acts as a master device, polling devices on the network and pushing the received data to the cloud. The gateway device was designed to support legacy protocols such as Modbus, BACnet, and HTTP RESTful interface devices. The developed software was evaluated with respect to RAM consumption under various stress tests and bandwidth utilization.

In [4], the authors proposed a fog-based IoT platform for smart building, which consists of five layers: end devices, network connectivity, fog processing, cloud processing, and security and privacy layer. The end devices include sensor nodes (temperature and humidity sensor, light sensor, PIR sensor, and accelerometer sensor) and actuator nodes (feedback action). The work focused on indoor ambience monitoring and occupancy monitoring. A prototype has been deployed and tested in a testbed room for door/window state detection, room occupancy detection, and room lighting sense and control. In [5], the authors proposed an IoT architecture for hybrid wind/PV/diesel/battery on a university campus. The proposed architecture consists of four layers: power layer, data acquisition layer, communication network layer, and application layer. The work is considered a case study on a university campus. However, the work focused on network modeling and simulation of the communication network layer for the hybrid energy system with respect to network topology, link capacity, and latency.

In [6], the authors presented a review of the concept of IoT and its potential application in smart buildings where the major components of the IoT system consist of devices/sensors, networks, cloud, analytics, and actuators/user interfaces. The conventional architecture for the smart building consists of three layers: the perception layer, network layer and application layer. The work discussed the challenges and recommendations for future research, including (1) security and privacy issues, (2) data acquisition, processing and storage issues, (3) feasibility and practicality issues, and (4) collaboration between IoT developers and the building industry. In [7], the authors presented a survey for different types of applications in smart buildings, including security control, energy management, monitoring and control of HVAC, water management, lighting system, fire detection, and health system for elders.

In [8], the authors proposed IoT based thermal model learning framework for a smart building based on low-cost IoT devices (smart thermostats). The data collected from the IoT platform installed inside the building has been used for validating the learning framework. In [9], the authors presented a low-cost solution for non-smart residential load appliances using smart load nodes. The integration of this solution does not require any change in the electric infrastructure of the house, as well as no modifications to the load appliances. The system considered wireless communication using WiFi in HAN, where the main measurements include voltage, current, power, and power factor.

In [10], the authors presented a comprehensive survey on the intersection of IoT and smart grid systems (IoT-aided smart grid systems), which includes architectures, applications, and prototypes. The work also presented different challenges and future research directions. In [11], the authors reviewed the architectures and functions of IoT-enabled smart energy grid systems. Special focus was given to IoT technologies such as sensing, communication, and computing. The work also reviewed security vulnerability, attack models, and existing threat summarizing mitigation techniques for such security vulnerabilities. In [12], the authors reviewed recent activities related to IoT-based energy systems. The work highlighted the potential areas to improve at different layers and reviewed communication technologies and standards related to energy systems. Some examples were discussed, including smart homes, smart power grids, and smart cities.

In [13], the authors presented a hierarchical IoT-based microgrid for energy-aware buildings. The proposed framework consists of the physical layer, information layer, control layer, and dispatch layer. The IoT microgrid laboratory at Aalborg university was introduced to explain how to implement the proposed scheme in a building. In [18], the authors presented a comprehensive review of thermal comfort in hospitals, identifying the current status of research and future research directions. The main research themes were influencing factors, field surveys, measures to improve and energy saving.

In [19], the authors proposed an IoT-based occupancy-driven plug load management system with the objective of reducing the energy consumption of plug loads and plug load automation. Six different strategies for plug load control, such as manual control, predefined schedule, occupancy-driven control, and hybrid control, were evaluated during a field study of 5-months within a university office space. In [22], the authors introduced the application of deep learning and IoT to control the operation of air conditioners to reduce energy consumption in a smart building. In order to count the number of persons in a certain area, the work considered the YOLOv3 algorithm. In [23], the authors provided architectural elements of connected indoor lighting systems within a building. In particular, application programming interfaces (APIs) were presented to support data access and lighting system control.

Table 1 summarizes the discussed related work highlighting the presence or absence of the different smart building IoT layers. The work proposed here aims to develop a hardware/software solution to enable gathering the information from smart sensors and energy monitoring devices and display it to the end users so they can take the necessary actions for the proper functioning of the building.

## 3. IoT-Based Architecture for Smart Buildings

### 3.1. Hierarchical Energy Management Architecture for Intelligent Campus

**Figure 1:**
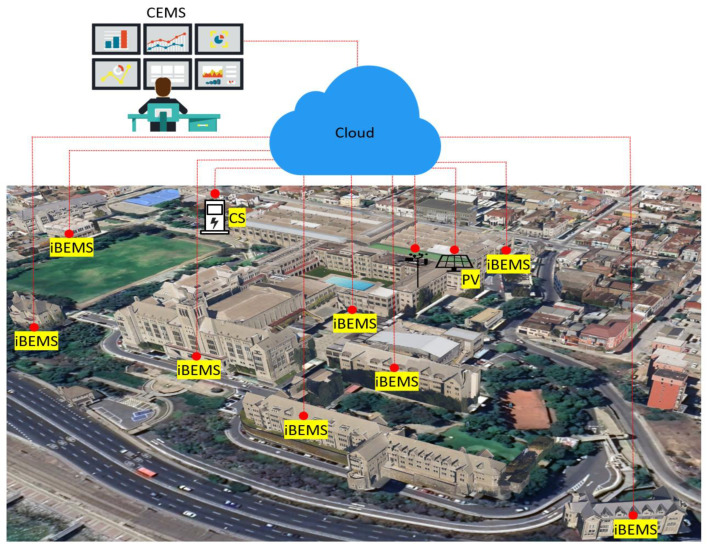
The schematic diagram for the intelligent campus. CEMS: campus energy management system; iBEMS: intelligent building energy management system; CS: charging station; PV: photovoltaic system.

A university campus generally consists of a group of buildings connected to the main power grid. The energy management approach can be classified into different levels: campus energy management system (CEMS), building energy management system (BEMS), and office/laboratory/classroom level. The CEMS operates as an energy manager for the campus by collecting energy consumption data from each building through intelligent BEMS (iBEMS), as shown in Figure 1.

- Campus energy management system (CEMS): The CEMS monitors the power generation (renewable energy), energy storage, and consumption of university buildings and interacts with each BEMS to optimize energy usage. In addition, the CEMS receives the energy consumption data of each building, stores it in a database, and estimates the consumption and future generation based on historical data.
- Building energy management system (BEMS): In general, university buildings consist of a group of offices, laboratories, and classrooms. The BEMS collects the energy data and other weather information collected by different smart meters and sensors located in the building and interacts with the building loads.

The CEMS provides large-scale data acquisition, communication, and data processing for energy management in buildings which requires cooperation among each BEMS in order to meet the operator requirements for minimizing power consumption and costs. However, as the number of energy management units increases, many challenges are related to cost, latency and reliability. This work target one building (Building B) of the UTFSM Campus, Valparaiso, Chile.

The university campus (Casa Central, Valparaíso, Chile) was inaugurated in 1931. Nowadays, there is a lack of monitoring and control for energy consumption in university buildings. There is still a high cost for building automation systems for small and medium-sized buildings, which prohibits purchasing such solutions. Furthermore, there is still a limitation for compatibility with different vendors, devices, and communication technologies in case of relying on using a particular platform. The proposed IoT platform aims to develop a custom solution using cost-effective off-the-shelf sensors/devices for real-time monitoring and control of building energy consumption through a web interface. Three case studies are considered: an office, a laboratory, and a classroom. Three case studies are considered: an office, a laboratory, and a classroom.

### 3.2. IoT-Based Architecture for Smart Buildings

**Figure 2:**
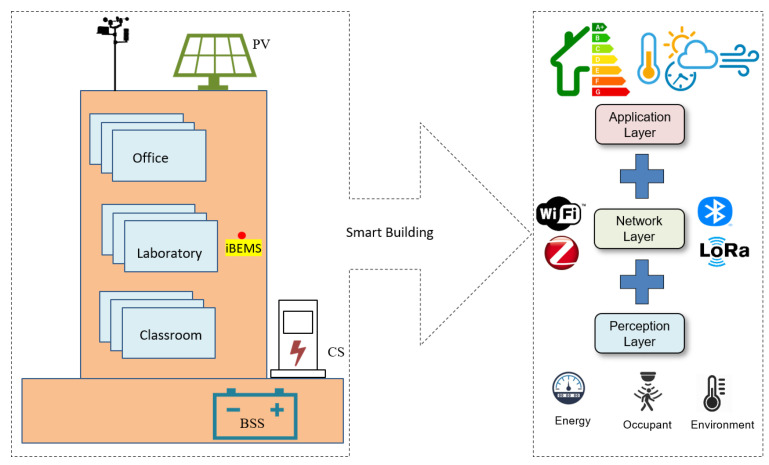
The schematic diagram for IoT-based architecture for smart buildings. iBEMS: intelligent building energy management system.

The main objective of this work is to design and implement an IoT platform for real-time monitoring and control of energy consumption in buildings to achieve an intelligent campus. There are different IoT architectures., such as three-layer (perception layer, network layer, and application layer), four-layer (perception layer, network layer, service layer, and application layer), and five-layer (perception layer, network layer, service layer, application layer, and business layer) [5,10,11,12,13,14]. Considering the various IoT-based architectures, Figure 2 shows the main components of a three-layers IoT-based architecture for smart buildings, which consist of the perception layer, network layer, and application layer. The perception layer usually emphasizes energy usage, occupant activities, and environmental condition. Different wired/wireless communication technologies could be used for data transmissions, such as WiFi, ZigBee, Bluetooth, and LoRa in the network layer. The application layer corresponds to business, application, and service management. The collected data at the application layer explains the actual building energy usage, energy management, occupant information, etc.

**Figure 3:**
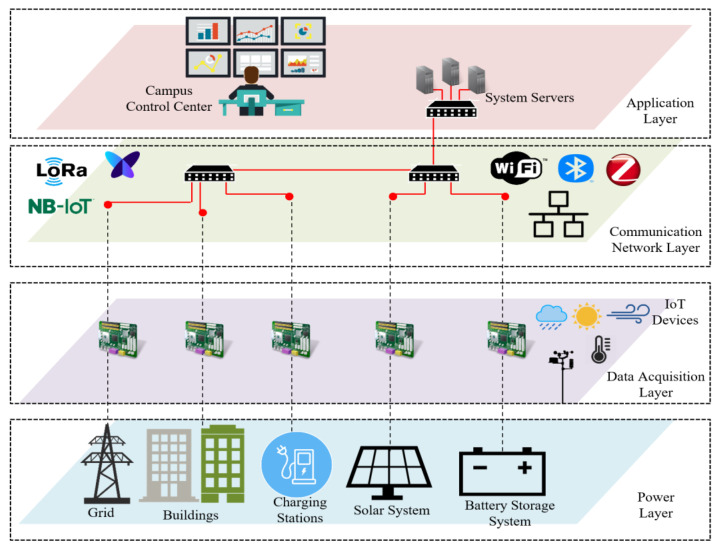
IoT-based architecture for the intelligent campus.

Figure 3 shows a detailed schematic diagram of the proposed IoT-based architecture for smart buildings. Our work aims to achieve building automation to minimize energy consumption costs and guarantee the comfort of the occupants. Our proposed architecture consists of four layers: power layer, data acquisition layer, communication network layer and application layer.

- Power Layer: the power layer consists of power generation, power storage, and loads that are connected to the power grid. In this study, the campus buildings are connected to the power distribution network (provided by Chilquinta). Other power generation sources may include solar panels, wind turbines, and batteries for power storage. The main power consumption in buildings may consist of HVAC, lights, and vehicle charging stations.
- Data Acquisition Layer: the data acquisition layer is responsible for capturing all the data coming from the power layer devices for making decisions. Examples of sensor nodes are measuring devices from light, temperature, power consumption, and meteorological station.
- Communication Network Layer: Different communication technologies and protocols are defined from data acquisition devices in the communication network layer. The communication network layer receives the sensors’ data and sends them to the application layer. Data might need to be sent over various networks, such as the local area network (LAN) and building area network (BAN). The most common communication technologies are ZigBee, Bluetooth, WiFi, and LoRa, using different communication protocols such as MQTT, CoAP, and Web Socket.
- Application Layer: the end-user can recognize the middleware services that allow data storage and interaction with building data in the cloud. All monitoring and status information received from the devices are stored and visualized. Real-time monitoring and control can then be achieved using different approaches such as energy management, safety, user comfort, and management of HVAC.

## 4. Smart Building Implementation

The proposed solution was implemented considering three locations with different needs. The details for each scenario and location are given below:

- Office Room: The first scenario is office B-349. This is a representation of professors’ offices distributed in Jeonju pus. Most offices include computers with one or more monitors, printers, and plugs to charge mobile devices. All offices include fixed lighting activated by a switch on the wall. Because it is a relatively small space, the energy measurement of the entire room was not considered.
- Laboratory Room: The second scenario is laboratory B-110. In this laboratory, there are at least four permanent workstations where a computer can be connected and external monitors for each of these positions. In addition, there is a shared space to carry out different activities. In this case, the implementation of smart plugs is proposed to monitor computers and other equipment connected to the power network. On the other hand, given the energy requirements of the space, it is suggested to install an energy meter in the electrical panel to monitor the total energy in the room.
- Classroom: The third scenario is classroom B-213. This classroom has luminaires that can be controlled with smart switches, one projector that can be controlled with smart plugs, and several sockets that allow students and teachers to connect their personal devices. An energy meter can be used on the electric board. In addition, an environmental measuring device can be installed to monitor the air quality during the classes.

**Figure 4:**
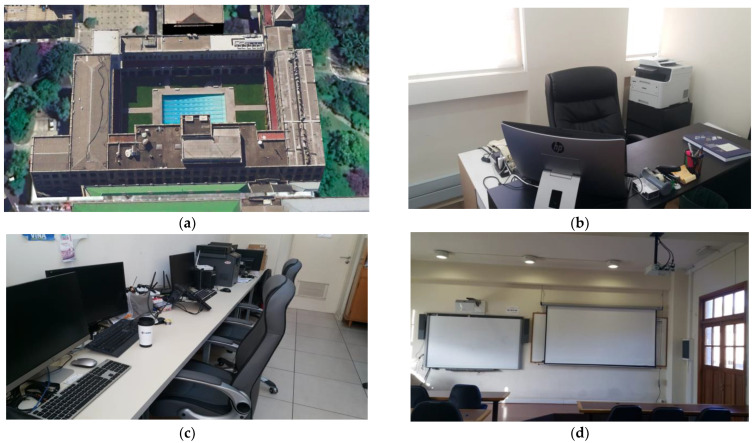
Locations of different implantation scenarios: (**a**) Engineering building B; (**b**) Office B-349; (**c**) Laboratory B-110; and (**d**) Classroom B-213.

Figure 4 shows the locations of different scenarios, while Table 2 shows the list of appliances and sensors considered at each location.

### 4.1. Selected Alternatives Solutions for Devices, Technologies, and Services

**Figure 5:**
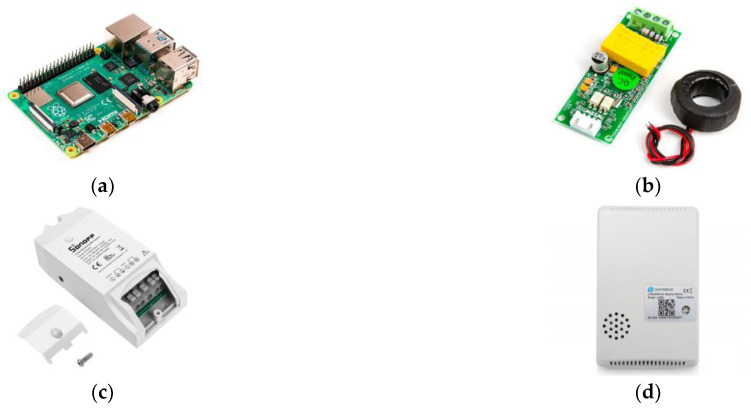
Selected devices for data acquisition layer: (**a**) Raspberry Pi 4B; (**b**) PZEM-004t-100 A; (**c**) Sonoff Pow R2; and (**d**) Air quality LAQ4.

**Figure 6:**
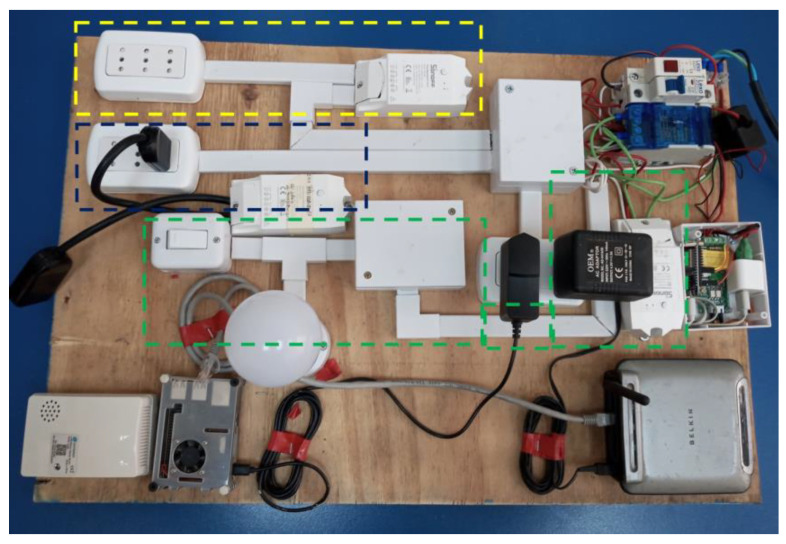
SThe schematic diagram for the testbed.

The implementation is carried out considering the proposed 4 layers discussed in the previous section. The description of equipment and technologies used in each layer are given in Table 3 and Figure 5. Although the system is proposed and designed for the three different scenarios in different locations, we organized and assembled a testbed shown in Figure 6. The testbed will allow us to collect data from various appliances individually and/or together for later using it to validate different machine learning algorithms.

The testbed is powered by an external source connected directly to a small electrical panel. On the board, there is a pilot light to verify the electric power at the input, then a switch for protection in case of current overconsumption, and a tetrapolar bar to facilitate the connection of equipment on the testbed. The testbed also includes two circuits: the lighting circuit and the sockets circuit. Furthermore, other components include a small router that provides a local WiFi network for the equipment, a LoRa gateway, and a Raspberry Pi that implements the proposed architecture. Detailed characteristics, setup and comparison among different types of sensors, measuring devices, and network elements are given in Appendix A and Appendix B.

### 4.2. Power Layer

#### 4.2.1. Monitoring Power Consumption of Appliances

For monitoring the power consumption of appliances, smart plugs of the “Sonoff POW R2” type are used [24]. All smart plugs were updated with ESPurna firmware and configured for wireless data transmission using WiFi [25]. Regarding the electric connection, the smart plugs need to be connected with electric cables and installed between the electric power supply and the appliances.

Different configurations for using smart plugs type “Sonoff POW R2” are considered for the testbed:

- Control the complete circuit: In this case, the whole lighting circuit was passing through the smart plug, which allows measuring and control of the lights (highlighted with green color in Figure 6)
- Control a socket: In this case, the connection was configured, which allows obtaining data from all equipment connected to the socket (highlighted with yellow color in Figure 6)
- Control a single device: This configuration allows a single device to be connected (highlighted with blue color in Figure 6)

#### 4.2.2. Monitoring Total Power Consumption

The total power consumption measurement is carried out using the pzem-004t-100a module. The module measures the input voltage of the electrical panel under study and a current transformer sensor for the current. The module is connected to the ESP32 platform for subsequent data sending.

#### 4.2.3. Monitoring Photovoltaic System

The main parameters considered for monitoring the photovoltaic panel include measuring the current with a current transform (CT) sensor. The sensor delivers a current proportional to the measurement current and a voltage divider for voltage measurement. Both measurements are captured with ADC pins of the Arduino nano development board. Data is sent to the NodeMCU development board Amica, which has WiFi communication and send the data by MQTT along with the date and time they were captured using a real-time clock (RTC).

### 4.3. Data Acquistion Layer

#### 4.3.1. Monitoring Indoor Environmental Condition

The air quality sensor type “Dragino LAQ4” was used to monitor indoor environmental conditions [26]. The main parameters measured are total volatile organic compound, CO2 equivalent, temperature, and relative humidity of the air. To obtain such indoor environmental data, the configuration of LoRa Gateway is required. In this work, we use the Dragino Gateway LG308 [27].

#### 4.3.2. Monitoring Weather Station

The meteorological information was measured using Davis Advantage Pro 2 Plus weather station [28]. The weather station obtains the data from the sensors physically connected to the station, then sends data wirelessly to the Vantage Pro 2 console. If connected to the datalogger, we can connect the console with a USB cable to the computer. Please note that the weather station is a closed system that does not allow the external manipulation of the data obtained.

### 4.4. Communication Network Layer

#### 4.4.1. Network Layer

The communication protocols for data transmission are MQTT and LoRa, which have been considered for implementing the communication network layer. For the smart plugs, the communication using MQTT is activated and configured by choosing the MQTT section from the side menu displayed in the web interface of the smart plugs with ESPurna firmware installed. In the case of general measurement devices and photovoltaic panels, the integration of the MQTT protocol is carried out within the code which we were programmed with. The configurations for all devices connecting via MQTT are similar; that is, they connect to the broker running on the Raspberry Pi with the IP 192:168:2:2 through port 1883 and are differentiated by the topic in which they publish. The topics were defined as a descriptive manner of the measurement and the locations where the data are taken and displayed, as shown in Table 4.

In the case of the air quality measurement device (LAQ4), a procedure should be carried out for the configurations. An account is created on the things network server at (https://www.thethingsnetwork.org (accessed on 10 January 2022)) [29]; then, when registering, you must enter the start section (which leads to the address https://console.cloud.thethings.network/ (accessed on 10 January 2022)) where the Cluster “Nam1” located in the state of Carolina in United States was chosen, and once selected you enter the Gateways tab where you press the + add gateway button, filling in all the requested data, in particular, the *Gateway ID* which is the unique number associated with each gateway LoRa. For the case of this work, the names used are *GatewayOfBuilding*, and the gateway ID is gatewaylorabuilding01. Once the registration of the LoRa gateway is completed, one can return to the gateways tab and select the registered gateway to see information about this connection with the network server.

Once the gateway connection has been verified, we go to the applications tab and press the button + add application. Only 3 parameters must be filled, namely “Application ID”, “Application Name” and “Description” that, for this case is filled with the data of smart-“buildingslora-sensors-usmcc” in Application ID and “lora sensors in usmcc” in Application Name. Once the application has been created and when entering it, press the button + add end device and enter the data for the desired sensor, which in this case is the LAQ4. Then, data can be entered corresponding to the manufacturer and model of the device. Next, the sensor’s own parameters, called Registration Key, are entered. In addition, in this case, the value of “airqualitysensorusmcc” was added to the parameter End Device ID, and Register End Device was pressed. Once registration is complete, go back to the applications tab, and in the end devices, one can enter the newly created one. Finally, being inside the added device, it is possible to enter the side menu section Integrations and then MQTT, where the subscription data to the broker of the things network, data that will be used in the application layer to visualize the data.

#### 4.4.2. Cloud Service

There are different service providers for the cloud layer (middleware). The major public service providers include Amazon, Microsoft, and Google [30,31]. Being leaders in the market, it is observed that the services are similar in almost all the services they provide. Due to the difficulty in calculating the costs associated with the service (the charge is made per hour of use and depends on the capabilities contracted), particularly when the project starts and the requirements can change; therefore, it was decided to use the service of virtual machines of Digital Ocean [32].

For this layer, a Raspberry Pi with the Raspberry Pi OS operating system is used, which is loaded into a micro-SD memory by downloading the installation software from (https://www.raspberrypi.com/software/ (accessed on 10 January 2022)), choosing the procedure to install and selecting the memory micro SD to use in the aforementioned Raspberry Pi. For later configuration, the system is accessed using a display with HDMI input and a mouse to activate the option to allow the connection by the SSH protocol. After doing this, it simply connects to the local network via the Ethernet cable. Then, on a computer within the same network, the connection is made via SSH (considering that the IP of the Raspberry Pi is 192.168.2.2).

**Figure 7:**
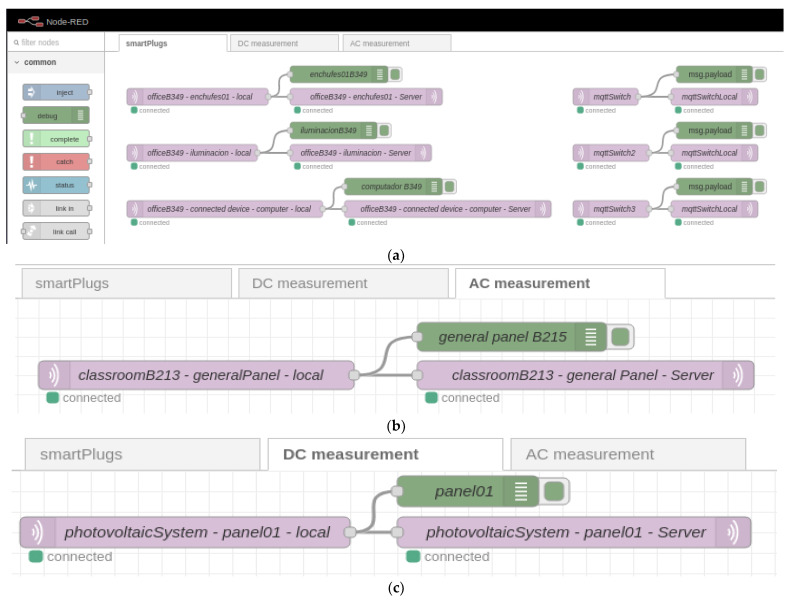
Node-RED configuration for forwarding data received from different devices (**a**) smart plugs; (**b**) general energy measurement; (**c**) direct current measurement.

Once the computer is connected to the Raspberry Pi via SSH, Mosquitto is installed as the selected MQTT broker [33]. Then, Node-RED is installed, with which the data is managed locally, following the official page’s recommendation (nodered.org). To access the node-red programming palette, it is enough to be within the same local network of the Raspberry Pi and go to http://192.168.2.2:1880. Then, once inside the programming palette, the division is made into 3 flows that correspond to smart plugs (see Figure 7a), the metering device alternating current (see Figure 7b) and the direct current measuring device (see Figure 7c). In the 3 flows, MQTT data is received from each device and forwarded using the same protocol (MQTT) to the server hosted at Digital Ocean.

### 4.5. Application Layer

**Figure 8:**
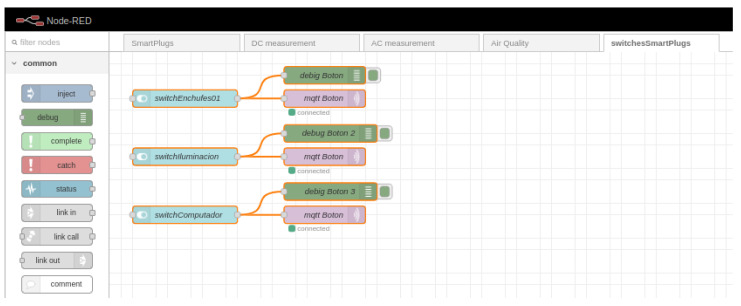
Configuration for Node-RED for the control of smart plugs.

**Figure 9:**
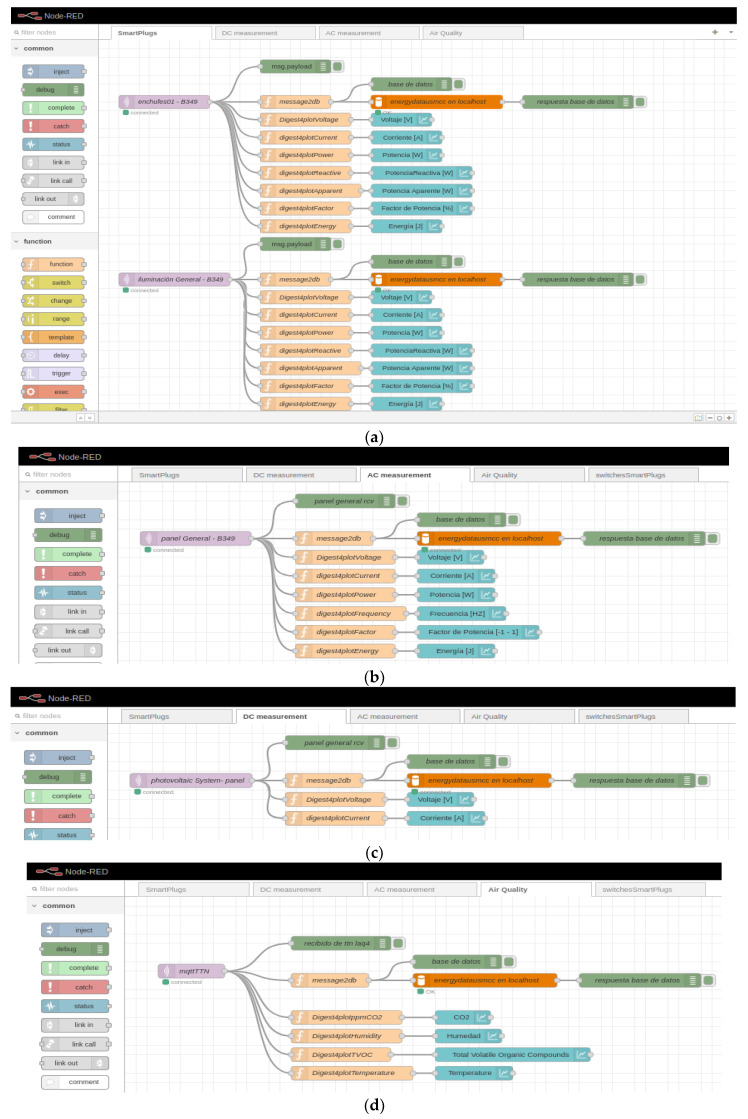
Configuration for Node-RED for data visualization: (**a**) Smart plugs; (**b**) Electric panel; (**c**) PV system; and (**d**) Air quality.

The application layer is entirely supported on a virtual machine contracted with the services of Digital Ocean. The system supports a machine with 2 Gb of RAM and 50 Gb of hard disk. To acquire these services, one must enter the provider’s page https://www.digitalocean.com/go/developer-brand (accessed on 10 January 2022) AND must be registered (considering that a valid credit card must be included during the registration process). After logging in to the page, a new project is generated, which, in this case, is called IoTPlatform4ManageEnergy and then the create button is pressed to create a new Droplet which is the way that Digital Ocean calls virtual machines, and this is created with 2 Gb of RAM, 50 Gb of hard disk and the Ubuntu 20.04 operating system is installed (LTS) x64. Once the Droplet has been created, it can be accessed with the fixed IP provided by the provider (165.232.139.50) via SSH. The Mosquitto broker was installed on this server to define communication via MQTT. In addition, Node-RED is installed to manipulate the data, and MySQL is installed to allow data storage. Figure 8 and Figure 9 show the configuration for Node-RED for control and data visualization, respectively.

**Figure 10:**
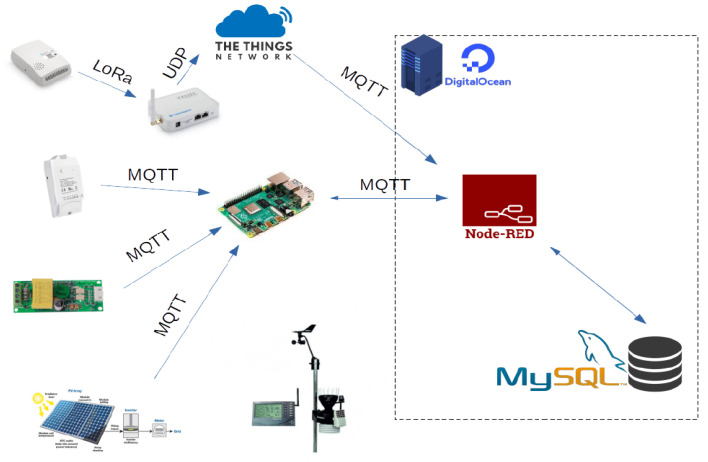
Schematic diagram for the complete system.

**Figure 11:**
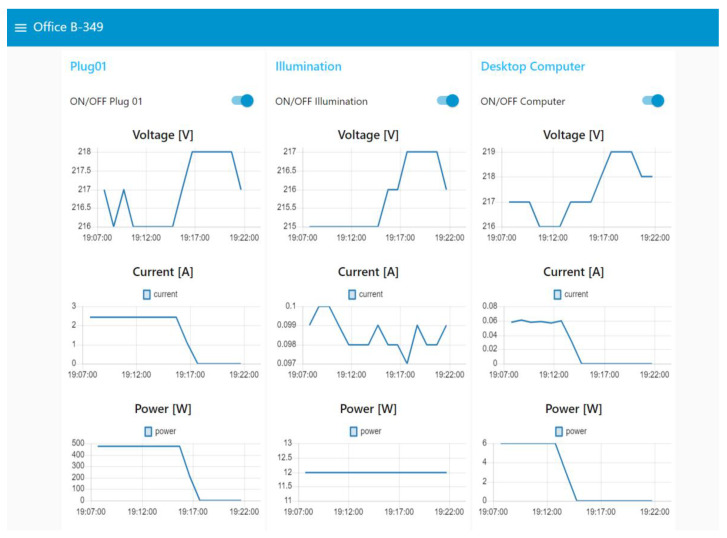
Real-time monitoring data from an office room.

**Figure 12:**
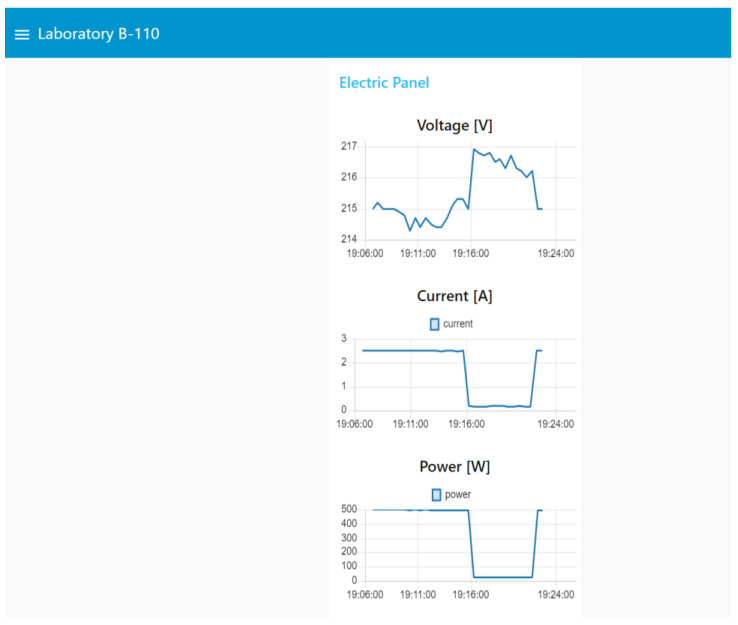
Real-time monitoring data from a laboratory room.

**Figure 13:**
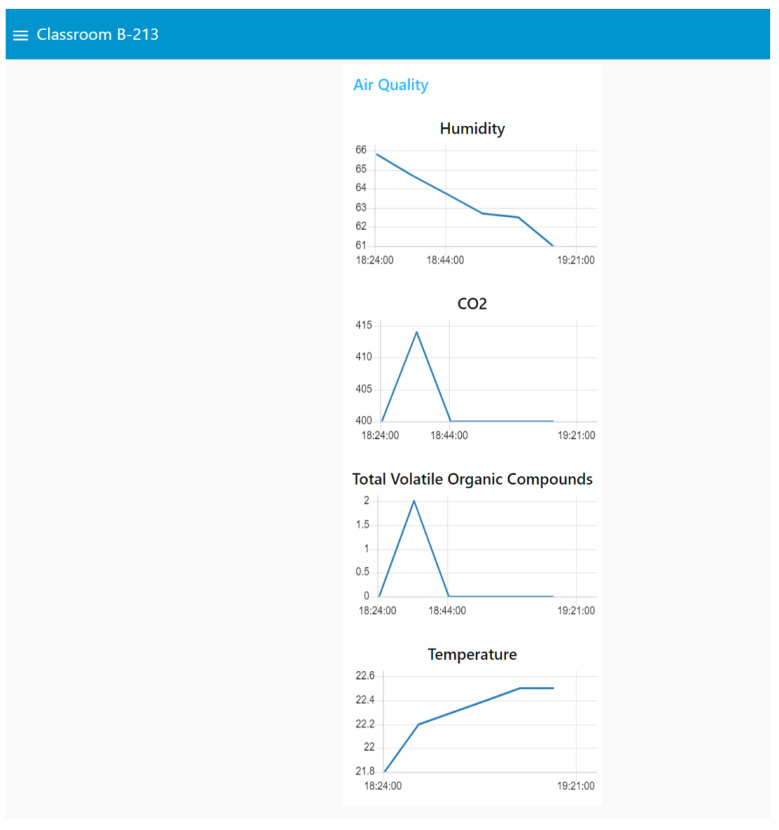
Real-time monitoring data from a classroom.

The schematic diagram for the complete system is shown in Figure 10. The experiments were carried out on 11 November 2022. Different electric appliances in the laboratory were connected to validate the operation of the platform (Plug01: Hair Drayer-SiEGEN-Model SG-3049, Illumination: Led bulb-9W, Desktop Computer: TV Monitor-LG-24TL520S-PS). The dashboard, shown in Figure 11, Figure 12 and Figure 13, shows an example for those observed from a computer; since Node-red is responsive, the visualization can adapt according to the display device, such as a smartphone or a tablet. Furthermore, with the implementation of the MYSQL database interaction block, the data is saved in the cloud.

## 5. Discussion

This section provides a detailed analysis of the proposed IoT platform for building implementation and the future extension for large-scale performance on the university campus.

### 5.1. Analysis

The advances in IoT technologies will play a vital role in the development of different smart building solutions, such as smart lighting to minimize light load, smart HVAC to improve indoor comfort, smart plug loads to monitor and control usage, and smart energy management systems, toward achieving an intelligent campus. Among the key technologies which have been investigated in this work are smart plug loads to monitor and control various types of appliances (located in offices, labs, and classrooms), distributed energy resources including a PV system, outdoor data using a meteorological station, and indoor air quality monitoring. Detailed technical hardware/software configuration and implementations have been discussed, as well as the network connectivity among different subsystems. As real-time data monitoring is the first step toward transforming conventional buildings into smart buildings, the proposed solution will enable the building operator to view, analyze, and predict different appliance profiles and the occupancy of the buildings using a dashboard to visualize such real-time/historical data and alerts.

With respect to the cost, the proposed solution uses off-the-shelf components. The main electronic components used were Sonoff POW R2, PZEN-004T-100A, Raspberry Pi 4B, and Dragino LAQ4, which cost about CLP 20.900, CLP 26.812, CLP 78.390, and CLP 51.899, respectively. The total hardware cost of the proposed solution can be adapted for the university campus to support real-time data monitoring for power consumption from different buildings and environmental data with the objective of improving energy performance and building operation.

Given the experience gained during the development of the proposed IoT platform, the following are the guidelines that need to be considered for developing such a solution (in Chile or another region) that fits the user’s requirements.

- Definition of requirements and services: This allows knowing the main problems and defining the objectives to be achieved during the full development of the proposed solution following the requirements:
- Define a network architecture for smart sensors and meters. Structure a network architecture that connects nodes, gateways, and servers seamlessly and efficiently to measure real-time electricity consumption and transmit the information obtained for the end-user.
- Develop a cloud storage server. This allows data storage and access to the information stored from anywhere through an API.
- Develop a platform for visualization. This platform enables the visualization of real-time data from each device/appliance connected to the platform.

### 5.2. Technology Adoption

The way toward an intelligent campus requires the acceptance of new technologies and the opportunities they provide. User perceptions on the acceptance and adoption of smart energy management in the workplace is an essential aspect that needs to be investigated [20,21,34]. In this regard, the work in Ref. [20] identified different types of behavior change interventions that are successful in saving energy in the workplace, such as education, persuasion, incentivization, and training. Another study in Ref. [21] proposed seven design implications that guide the future design of smart energy management systems in the workplace, including internal and external influence, user appeal, user control, ease of use, reliability, personalized and contextualized information, and data privacy. Among the open research topics are conducting a large-scale study in multiple countries (different geographical contexts and cultures) to identify overlaps in user perceptions. In addition, expanding the scope for other workplaces such as hotels, retail stores, and industrial sites [21].

### 5.3. Future Direction

There are different directions to extend the current work. From the power layer point of view, we aim to extend the current prototype to support an electric vehicle charging station, a battery storage system, smart lighting (dimmer lights and lights coupled with motion sensors), sensors for motion detection, and magnetic sensors for the doors. From the data acquisition layer point of view, our ongoing work aims to develop a low-cost meteorological station using off-the-shelf components to be able to access and control the data obtained. From the communication network layer point of view, heterogeneous communication technologies could be integrated to support different short-/long-range communications across the campus.

As a short-term goal, we aim to demonstrate the feasibility of the proposed IoT platform in one complete building to collect power consumption data during the whole semester with everyday activities and during the vacation period. Before implementing such a system, two different dashboards need to be developed: one for the end-user, who can control their plug loads, and the other for the building administrator. There is also a need to define which control strategy should be selected, which can be a manual or predefined schedule. In this regard, the authors in Ref. [19] performed different plug load automation strategies for a university office space which could be a starting point for the office scenario.

Other challenges are related to the internal electrical wiring for the building, as each floor may share various offices and laboratories from different departments. In addition, the absence of historical power consumption data for buildings/floors/zones. Therefore, historical power consumption data will need to be collected from the main electric panels using smart meters for individual rooms/zones. This step is essential to define spaces with high energy consumption. Based on energy consumption data from different floors/areas, the performance will be evaluated before and after applying different plug load management strategies. Special attention should be given to privacy and security, which should be considered at every step during the platform design. Emerging technologies such as blockchain, machine learning, and artificial intelligence are opening new opportunities to mitigate such security vulnerabilities.

## 6. Conclusions

This work proposed a cost-effective IoT solution for smart buildings to enable remote monitoring and control of power consumption at the appliance level. The proposed architecture consists of four layers: power layer, data acquisition layer, communication network layer, and application layer. The physical layer was characterized by different subsystems such as plug circuits, lighting, and the photovoltaic system. For the acquisition layer, measurement devices were used for electrical panels, smart plugs, direct current energy metering devices, air quality monitoring, and a meteorological station. The network layer was defined to gather the information captured from the physical layer and forward it to the remote server. A fog layer was implemented on a Raspberry Pi, and the data was handled with NodeRED. The communication technologies defined to obtain the information from the installed equipment were WiFi and LoRa, and the communication protocol to the server was through the MQTT. The Digital Ocean Droplet service was used as a server where the MQTT Broker was installed. For data management, NodeRED was installed for the general management of the data messages and the visualization by the end user. MySQL was installed, which allows storing the information in tables that were defined for each data acquisition device. This project was implemented using a testbed defined to characterize equipment and conditions in three different locations in Engineering Building, Universidad Técnica Federico Santa María, Valparaíso, Chile, including an office, a classroom, and a laboratory. This testbed allowed the design, implementation, and testing of the complete system in reduced space. At the end of the development of this work, a functional platform was obtained that brings together energy consumption data that will contribute to energy awareness and conservation.

## Figures and Tables

**Table 1:** Summary of related work for different IoT layers in related work for smart buildings.

Ref.	Type/ Case Study	Power Layer	DAQ Layer	Network Layer	Application Layer	Contribution
[2]	Technical/ Australia	YES	NO	NO	YES	Building energy management with key insight into human activity and occupancy detection.
[3]	Technical/ USA	NO	YES	YES	NO	Building energy management with a focus on the software architecture and software design for the gateway device to support various legacy protocols.
[4]	Technical/ Egypt	NO	NO	YES	YES	Fog-based IoT platform consists of five layers: end devices, network connectivity, fog processing, cloud processing, and security and privacy layer. The work focused on indoor ambience monitoring and occupancy monitoring.
[5]	Simulation/ Saudi Arabia	NO	NO	YES	NO	The work focused on network modeling and simulation of the communication layer for a hybrid energy system with respect to network topology, link capacity and latency.
[6]	Review	YES	YES	YES	NO	Overview of IoT technology for smart buildings. The components of the IoT system are devices/sensors, networks, cloud, analytics, and actuators/user interfaces.
[7]	Review	YES	NO	NO	YES	Survey on different types of applications in the smart building, including security control, energy management, monitoring and control of HVAC, water management, lighting system, fire detection and health system of elders.
[8]	Technical/ USA	YES	NO	NO	YES	IoT-based thermal model learning framework for smart buildings based on low-cost IoT devices (smart thermostats).
[9]	Technical/ India	YES	YES	YES	NO	A low-cost solution for non-smart residential load appliances using smart load nodes (SLN).
[10]	Review	YES	YES	YES	YES	A comprehensive survey on the intersection of IoT and smart grid systems (IoT-aided smart grid systems)
[11]	Review	YES	YES	YES	YES	Review the architectures and functions of IoT-enabled smart energy grid systems
[12]	Review	YES	YES	YES	YES	Review for recent activities related to IoT-based energy systems. Examples were discussed, including smart homes, smart power grids, and smart cities
[13]	Technical/ Denmark	YES	YES	YES	YES	A hierarchical IoT-based microgrid for energy-aware buildings
[18]	Review	YES	NO	NO	NO	A comprehensive review on thermal comfort in hospitals, identifying the current status of research and future research directions.
[19]	Technical/ Singapore	YES	YES	YES	YES	IoT-based occupancy-driven smart plug load management system that reduces plug load energy consumption. The occupancy information of users is collected using an indoor localization system.
[22]	Technical/ Finland	NO	NO	NO	YES	Application of deep learning and IoT to control the operation of air conditioners to reduce energy consumption in a smart building.
[23]	Technical/ Netherlands	YES	NO	NO	YES	Architectural elements of connected indoor lighting systems within a building. In particular, APIs were presented to support data access and lighting system control
Present Work	Technical/ Chile	YES	YES	YES	YES	Developed a hardware and software platform for remote monitoring and control of smart buildings. A real testbed has been designed, implemented, and tested at Universidad Técnica Federico Santa María, Valparaiso, Chile.

**Table 2:** List of appliances.

Location	Details
Office Room (OR)	1 Computer, 1 Monitor, 1 Printer Lighting
Classroom (CR)	1 Projector, Many sockets Main electric board Lighting
Laboratory (LAB)	4 computers, 1 Printer, Many sockets, Lighting PV panels

**Table 3:** List of sensors and measuring devices used for the testbed.

Layer	Details
Data Acquisition Layer	Smart Plug, Air quality sensor, Smart meter for the total power consumption, Data acquisition module for photovoltaic system, Data collection module for a weather station
Communication Network Layer	Cloud Service (Raspberry Pi, Node-RED) Network Layer (WiFi, LoRa, MQTT)
Application Layer	Digital Ocean, Node-RED, MySQL

**Table 4:** Description of topics connected to MQTT.

Name	Task	Location	Topic
Sonoff POW R2	Smart Plug	Office B-349	officeB349/enches01/ ESPURNAA9F0E4
Sonoff POW R2	Illumination	Office B-349	officeB349/ilminacionGeneral ESPURNA9CFBF8
Sonoff POW R2	Computer	Office B-349	officeB349/connectedDevice/Computer/ESPURNA9CFBF8
DC Measurement	PV Panel	Outside	photovoltaicSystem/panel01
AC Measurement	General Electric Panel	Class B-213	classroomB213/generalPanel

**Figure A1:**
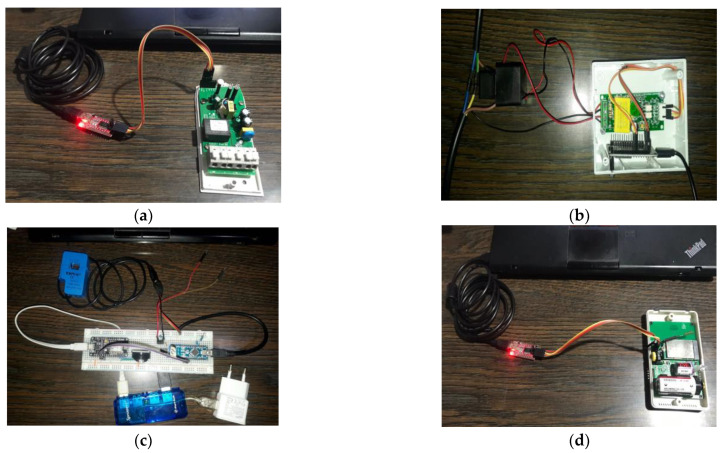
Setup of sensors and measuring devices: (**a**) smart plug device; (**b**) pzem-004t-100a module for total power consumption; (**c**) monitoring the PV system; and (**d**) Dragino LAQ4 for indoor environmental data.

**Figure A2:**
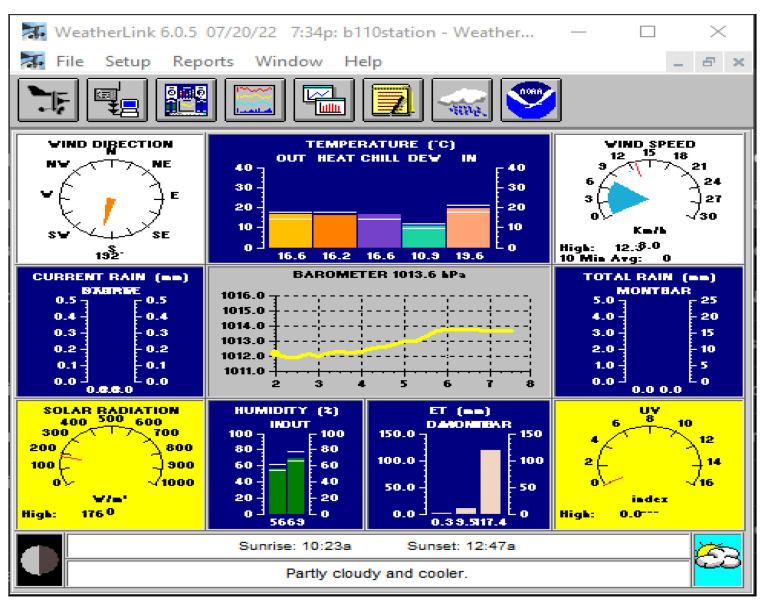
Weather station data obtained using Weather Link software.

## Data Availability

Not applicable.

## Nomenclature

IoT	Internet of Things
EMS	Energy Management System
BEMS	Building Energy Management System
CEMS	Campus Energy Management System
HVAC	Heating, Ventilation, and Air Conditioning
HTTP	Hypertext Transfer Protocol
REST	Representational State Transfer
WiFi	Wireless Fidelity
HAN	Home Area Network
SLN	Smart Load Node
CEMS	Campus Energy Management System
CS	Charging Station
PV	Photovoltaic
LoRa	Long Range
BAN	Building Area Network
MQTT	Message Queuing Telemetry Transport
CoAP	Constrained Application Protocol
TTN	The Things Network
CT	Current Transformer
ADC	Analog to Digital Converter
RTC	Real Time Clock
PIR	Passive InfraRed

## Appendix A

For all devices considered, the communication technology should be compatible with data acquisition, and great importance should be given to equipment and devices that are sold in the country (Chile). Table A1 shows the characteristics of the smart plugs. Due to their significant similarity, the only one that allows the power measurement of the device connected to it has been selected (Sonoff Pow R2). On the other hand, it is decided to use the same smart plug but connected in series to the lighting circuit.

**Table A1:** Characteristics of smart plug devices.

Model	Xiaomi Smart Plug *	Sonoff Pow R2 Smart Plug **	S26 WiFi R2 Smart Plug ***
Maximum current	16 A	16 A	16 A
Energy measurement	NO	YES	NO
Connectivity	WiFi 2.4 GHz	WiFi 2.4 GHz	WiFi 2.4 GHz
Price	19.990 CLP	20.990 CLP	14.990 CLP

* https://www.paris.cl/mi-smart-plug-wifi-MKA3SGOLRU.html (accessed on 22 October 2022); ** https://www.paris.cl/interruptor-wifi-diy-sonoff-pow-r2-MK4BFC5Y9J.html (accessed on 22 October 2022); *** https://www.paris.cl/sonoff-s26-r2/ (accessed on 22 October 2022).

For the energy measurement device, pzem-004t-100a equipment has been selected, as shown in Table A2. In the case of the temperature and humidity measurement device, significant similarities are observed in the characteristics of all the proposed alternatives. Dragino LAQ4 has been selected, which reads the most number of variables, as shown in Table A3. Table A4 shows the alternative gateway that allows obtaining the data from the LoRa sensors, where minor differences are observed between each device. Since the LG308 LORAWAN gateway is available in the laboratory, it has been selected for this work.

**Table A2:** Characteristics of energy metering devices.

Model	Sonoff POW R3 *	Vbestlife ZMAi-90 **	PZEM ***
Voltage input/output	100–240 V	90–250 V	80–260 V
Maximum current	25 A	60 A	100 A
Price	48.990 CLP	38.570 CLP	26.812 CLP

* https://www.paris.cl/interruptor-wifi-de-alta-potencia-sonoff-powr3-MKSS3WI6FZ.html (accessed on 22 October 2022); ** https://articulo.mercadolibre.cl/MLC-1069867809-medidor-de-energia-inteligente-wifi-de-consumo-unico (accessed on 22 October 2022); *** https://articulo.mercadolibre.cl/MLC-915082915-pzem-004t-voltaje-de-corriente-multimetro-modulo-80-260v-100 (accessed on 22 October 2022).

**Table A3:** Characteristics of devices with ambient sensors.

Model	LHT65 *	Sonoff SNZB-02 **	Sonoff TH16+AM2301 ***	Dragino LAQ4 ****
Parameters	Temperature Humidity	Temperature Humidity	Temperature Humidity	CO_2_, Temperature Relative Humidity
Power	Battery 2400 mAh	CR2450—3V	Power connected	4000mAh Li-SOC12
Connectivity	LoRa	ZigBee	WiFi 2.4 GHz	LoRa
Price	37.890 CLP	9.890 CLP	14.990 CLP	51.899 CLP

* https://altronics.cl/sensor-lht65-lorawan?search=LHT65 (accessed on 22 October 2022); ** https://altronics.cl/sensor-temp-hum-zigbee-sonoff (accessed on 22 October 2022); *** https://sonoff.cl/pack-interruptor-diy-sonoff-th16-sensor-de-temperatura-y-humedad-am2301/ (accessed on 22 October 2022); **** https://altronics.cl/sensor-calidad-aire-laq4?search=LAQ4 (accessed on 22 October 2022).

**Table A4:** Characteristics of LoRa gateway.

Model	LG308 *	Laird RG191 **	LIG16-915 ***
Transceiver	SX1308/SX1276	SX1301/SX1257	SX1302
Frequency	915 MHz	915 MHz	915 MHz
WiFi Connectivity	WiFi 2.4 GHz	WiFi 2.4 GHz	WiFi 2.4 GHz
Price	249.900 CLP	499.990 CLP	154.690 CLP

* https://altronics.cl/lora-gateway-lg308 (accessed on 22 October 2022); ** https://mcielectronics.cl/shop/product/gateway-lora-915mhz-wi-fi-bluetooth-ethernet-laird-rg191-laird-25707/ (accessed on 22 October 2022); *** https://altronics.cl/lig16-gateway-lorawan (accessed on 22 October 2022).

The proposed alternatives for the local computing device with its main hardware characteristics are summarized in Table A5. Considering the highest percentage of the connectivity item and the cheap cost of the device, the Raspberry Pi 4B was chosen as the local computing device.

**Table A5:** Characteristics of computing devices.

Name	Core/RAM	Storage	Connectivity	Price
Raspberry Pi 4B *	4/4-8	SD	Gigabit Ethernet WiFi, BLE	78.390 CLP
Odroid-xu4 **	8/2	Flash board	Gigabit Ethernet	99.990 CLP
Jetson Nano Nvidia ***	4/4	microSD	Gigabit Ethernet	185.990 CLP

* https://altronics.cl/raspberry-pi-4-4gb (accessed on 22 October 2022); ** https://mcielectronics.cl/?s=Odroid-xu4&post_type=product&product_cat=0, (accessed on 22 October 2022); *** https://mcielectronics.cl/shop/product/kit-de-desarrollo-jetson-nano-nvidia-nvidia-27557/ (accessed on 22 October 2022).

## Appendix B

Figure A1a shows the setup of the smart plug device, while Figure A1b shows the setup of the module pzem-004t-100a. Figure A1c shows the configuration of the solar panel measurement device. The CT sensor must be connected by wrapping the positive cable of the panel, and the terminals of voltage test (brown → ground and red → VCC) are connected in parallel to the current bus of the photovoltaic panel system. Figure A1d shows the configuration of the Dragino LAQ4 device. Figure A2 shows the data obtained using the weather station.

## References

[1] Energy Efficiency Law and Plan, Ministry of Energy. https://energia.gob.cl/ley-y-plan-de-eficiencia-energetica.

[2] Tushar W., Wijerathne N., Li W.-T., Yuen C., Poor H.V., Saha T.K., Wood K.L. (2018). IoT for Green Building Management. arXiv.

[3] Nugur A., Pipattanasomporn M., Kuzlu M., Rahman S. (2019). Design and Development of an IoT Gateway for Smart Building Applications. IEEE Internet Things J..

[4] Alsuhli G., Khattab A. A Fog-based IoT Platform for Smart Buildings. Proceedings of the 2019 International Conference on Innovative Trends in Computer Engineering (ITCE).

[5] Eltamaly A.M., Alotaibi M.A., Alolah A.I., Ahmed M.A. (2021). IoT-Based Hybrid Renewable Energy System for Smart Campus. Sustainability.

[6] Jia M., Komeily A., Wang Y., Srinivasan R.S. (2019). Adopting Internet of Things for the development of smart buildings: A review of enabling technologies and applications. Autom. Constr..

[7] Vijayan D.S., Rose A.L., Arvindan S., Revathy J., Amuthadevi C. (2020). Automation systems in smart buildings: A review. J. Ambient Intell. Humaniz. Comput..

[8] Zhang X., Pipattanasomporn M., Chen T., Rahman S. (2020). An IoT-Based Thermal Model Learning Framework for Smart Buildings. IEEE Internet Things J..

[9] Singh S., Roy A., Selvan M.P. (2019). Smart Load Node for Nonsmart Load Under Smart Grid Paradigm: A New Home Energy Management System. IEEE Consum. Electron. Mag..

[10] Saleem Y., Crespi N., Rehmani M.H., Copeland R. (2019). Internet of Things-Aided Smart Grid: Technologies, Architectures, Applications, Prototypes, and Future Research Directions. IEEE Access.

[11] Abir S.M.A.A., Anwar A., Choi J., Kayes A.S.M. (2021). IoT-Enabled Smart Energy Grid: Applications and Challenges. IEEE Access.

[12] Shakerighadi B., Anvari-Moghaddam A., Vasquez J., Guerrero J. (2018). Internet of Things for Modern Energy Systems: State-of-the-Art, Challenges, and Open Issues. Energies.

[13] Wu Y., Wu Y., Guerrero J., Vasquez J., Palacios-García E., Guan Y. (2020). IoT-enabled Microgrid for Intelligent Energy-aware Buildings: A Novel Hierarchical Self-consumption Scheme with Renewables. Electronics.

[14] Choi J.S. (2019). A Hierarchical Distributed Energy Management Agent Framework for Smart Homes, Grids, and Cities. IEEE Commun. Mag..

[15] Aliero M.S., Asif M., Ghani I., Pasha M.F., Jeong S.R. (2022). Systematic Review Analysis on Smart Building: Challenges and Opportunities. Sustainability.

[16] Fanti M.P., Mangini A.M., Roccotelli M., Ukovich W. (2015). A District Energy Management Based on Thermal Comfort Satisfaction and Real-Time Power Balancing. IEEE Trans. Autom. Sci. Eng..

[17] Plageras A.P., Psannis K.E., Stergiou C., Wang H., Gupta B.B. (2018). Efficient IoT-based sensor BIG Data collection–processing and analysis in smart buildings. Futur. Gener. Comput. Syst..

[18] Yuan F., Yao R., Sadrizadeh S., Li B., Cao G., Zhang S., Zhou S., Liu H., Bogdan A., Croitoru C. (2022). Thermal comfort in hospital buildings—A literature review. J. Build. Eng..

[19] Tekler Z.D., Low R., Yuen C., Blessing L. (2022). Plug-Mate: An IoT-based occupancy-driven plug load management system in smart buildings. Build. Environ..

[20] Staddon S.C., Cycil C., Goulden M., Leygue C., Spence A. (2016). Intervening to change behaviour and save energy in the workplace: A systematic review of available evidence. Energy Res. Soc. Sci..

[21] Tekler Z.D., Low R., Blessing L. (2022). User perceptions on the adoption of smart energy management systems in the workplace: Design and policy implications. Energy Res. Soc. Sci..

[22] Elsisi M., Tran M.-Q., Mahmoud K., Lehtonen M., Darwish M.M.F. (2021). Deep Learning-Based Industry 4.0 and Internet of Things towards Effective Energy Management for Smart Buildings. Sensors.

[23] Pandharipande A., Zhao M., Frimout E. (2019). Connected Indoor Lighting Based Applications in a Building IoT Ecosystem. IEEE Internet Things Mag..

[24] SONOFF Smart Plug Products. https://sonoff.tech/products/.

[25] ESPurna Firmware. https://github.com/xoseperez/espurna.

[26] LoRaWAN Air Quality Sensor. https://www.dragino.com/products/lora-lorawan-end-node/item/174-laq4.html.

[27] LG308 Indoor LoRaWAN Gateway. https://www.dragino.com/products/lora-lorawan-gateway/item/140-lg308.html.

[28] Davis 6152 Wireless Vantage Pro2. https://www.davisinstruments.com/pages/vantage-pro2.

[29] The Things Network. https://www.thethingsnetwork.org/.

[30] Liu Y., Akram Hassan K., Karlsson M., Pang Z., Gong S. (2019). A Data-Centric Internet of Things Framework Based on Azure Cloud. IEEE Access.

[31] Pierleoni P., Concetti R., Belli A., Palma L. (2020). Amazon, Google and Microsoft Solutions for IoT: Architectures and a Performance Comparison. IEEE Access.

[32] Digital Ocean. https://www.digitalocean.com/,.

[33] Eclipse Mosquitto. https://mosquitto.org/,.

[34] Krishnamurti T., Schwartz D., Davis A., Fischhoff B., de Bruin W.B., Lave L., Wang J. (2012). Preparing for smart grid technologies: A behavioral decision research approach to understanding consumer expectations about smart meters. Energy Policy.

